# Human 14-3-3 gamma protein results in abnormal cell proliferation in the developing eye of *Drosophila melanogaster*

**DOI:** 10.1186/1747-1028-3-2

**Published:** 2008-01-14

**Authors:** Sophia W Hong, Wenqing Qi, Marc Brabant, Giovanni Bosco, Jesse D Martinez

**Affiliations:** 1 Department of Cell Biology and Anatomy, Arizona Cancer Center, University of Arizona, Tucson, Arizona 85724, USA; 2 Department of Medicine, Arizona Cancer Center, University of Arizona, Tucson, Arizona 85724, USA; 3 Cancer Center Division, Arizona Cancer Center, University of Arizona, Tucson, Arizona 85724, USA; 4 Department of Molecular and Cellular Biology, University of Arizona, Tucson, Arizona 85721, USA

## Abstract

**Background:**

14-3-3 proteins are a family of adaptor proteins that participate in a wide variety of cellular processes. Recent evidence indicates that the expression levels of these proteins are elevated in some human tumors providing circumstantial evidence for their involvement in human cancers. However, the mechanism through which these proteins act in tumorigenesis is uncertain.

**Results:**

To determine whether elevated levels of 14-3-3 proteins may perturb cell growth we overexpressed human 14-3-3 gamma (h14-3-3 gamma) in Drosophila larvae using the heat shock promoter or the *GMR-Gal4 *driver and then examined the effect that this had on cell proliferation in the eye imaginal discs of third instar larvae. We found that induction of h14-3-3 gamma resulted in the abnormal appearance of replicating cells in the differentiating proneural photoreceptor cells of eye imaginal discs where h14-3-3 gamma was driven by the heat shock promoter. Similarly, we found that driving h14-3-3 gamma expression specifically in developing eye discs with the *GMR-Gal4 *driver resulted in increased numbers of replicative cells following the morphogenetic furrow. Interestingly, we found that the effects of overexpressing h1433 gamma on eye development were increased in a genetic background where *String *(cdc25) function was compromised.

**Conclusion:**

Taken together our results indicate that h14-3-3 gamma can promote abnormal cell proliferation and may act through Cdc25. This has important implications for 14-3-3 gamma as an oncogene as it suggests that elevated levels of 14-3-3 may confer a growth advantage to cells that overexpress it.

## Background

The 14-3-3 proteins are found abundantly in cytoplasm of brain neuronal cells [1,2] and are highly conserved in organisms as diverse as yeast, *Drosophila*, and humans [3,4]. Only two isoforms, ε and ζ are expressed in Drosophila [4] and yeast [5,6]. However, in mammals, there are seven family members and each is designated with a Greek letter (ε, γ, η, σ, θ/τ). Phosphorylated isoforms of β and ζ, respectively, are known as ΰ and δ [7]. All 14-3-3 family members have been shown to function in various aspects of crucial cellular processes including cell cycling regulation [8,9], apoptosis [10,11], transcriptional regulation [12,13] and Ras/Raf signaling [14].

The diversity of activities in which 14-3-3 proteins act is due to their ability to interact with a wide variety of signaling molecules through a variety of consensus motifs that typically consist of a phosphoserine residue flanked by an arginine and proline such as RXY(F)XpS(pT)XP and RSxpS(pT)xP (x stands for any amino acid, and pS refers to phosphorylated Serine), but may also bind to motifs that are serine-rich or to apparently unrelated motifs such as GHSL and WLDLE [3,15]. An added complexity is that 14-3-3s form thermodynamically stable dimers and each family member has a distinct preference for formation of either homo- or hetero-dimers providing a diversity of architectures for protein interactions [6]. For instance the γ protein forms homodimers as well as having a heterodimeric formation with the ε protein [6]. Conversely, the ε protein does not homodimerize, and instead prefers to heterodimerizes with other family members (η, β, γ, ζ) [6]. As a consequence, 14-3-3 proteins can regulate and/or influence the activity of a wide variety of proteins which accounts for their involvement in such a wide range of normal cellular processes.

Perhaps the best characterized cellular process that 14-3-3 is involved in is the ability to regulate cell cycle progression [16]. Detailed studies in yeast show that 14-3-3 binds to the key cell cycle regulator, Cdc25, in response to DNA damage which leads to Cdc25 being exported from the nucleus [17]. This checkpoint activation results in cells halting their entry into mitosis which facilitates the repair of DNA damage [17]. 14-3-3 proteins ε and γ play a similar role in regulating G2/M progression in humans [9,16,18]. Moreover, 14-3-3ζ was also shown to bind with Cdc25C in A549 lung cancer cells after irradiation [19]. Collectively, these studies show that 14-3-3 proteins play a role in maintaining genomic integrity.

The involvement of 14-3-3 proteins in cellular process that may be relevant to their role in human cancer is not limited to regulation of cell cycle checkpoints nor are ε and ζ the only family members that could have a role in tumorigeneis. For instance, exogenous expression of 14-3-3β increases proliferation of NIH3T3 cells and confers the ability to grow in soft agar [20]. 14-3-3θ was shown to induce the expression of tenascin-C (overexpressed in most solid tumors) which increase cell adhesion of mammalian MCF-7 carcinoma cells on a substratum [21]. Moreover, the expression levels of most 14-3-3s are elevated in lung and other cancers suggesting that they confer a growth advantage to neoplastic cells [22].

In these studies we chose to focus on the 14-3-3γ protein because we found that this family member was consistently upregulated in human lung cancers and when introduced into H322 lung cancer cells caused polyploidization suggesting that it might have potential oncogenicity [22,23]. Because flies have two 14-3-3 proteins that act on the same signaling pathways and cellular processes in human cells that are involved in carcinogenesis we chose Drosophila for our model system [4,24-27]. Consequently we utilized this genetically tractable model organism to examine the effect that targeted overexpression of h14-3-3γ had on cell cycling in the developing eye and found that 14-3-3γ stimulated abnormal cell proliferation in neuronal cells of the differentiating eye imaginal discs. We also examined genetic interactions between *String *(Drosophila Cdc25C homolog) and h14-3-3γ in terms of cell cycling regulation in fly eyes.

## Results

### Overexpression of human 14-3-3γ leads to abnormal cell proliferation in differentiating eye imaginal discs

**Figure 1:**
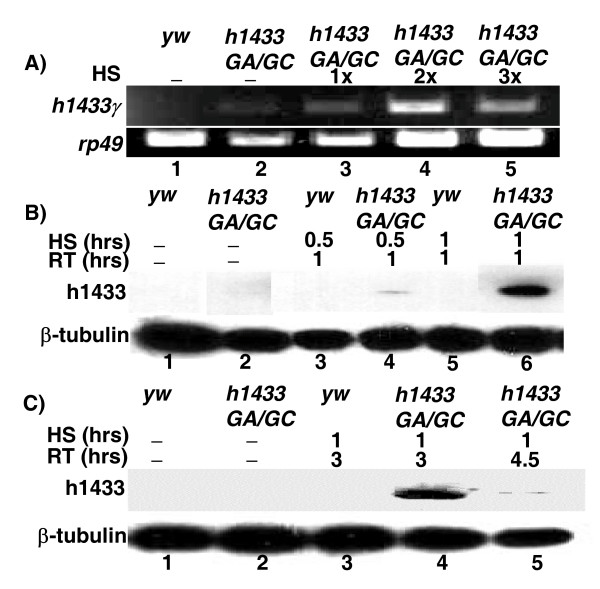
**Overexpression of human 14-3-3γ in Drosophila using the Hsp70 promoter**. (A) Third instar larvae harboring the human 14-3-3γ driven by the heat shock inducible Hsp70 promoter were collected after incubating at 37°C for one hour and the heat shock applied either once or multiple times as indicated in the figure. Total RNA was extracted and RT-PCR conducted using primers specific for the human 14-3-3γ RNA and the PCR products electrophoresed on agarose gels. *Yw, yw*^67*C*2^, was used as a control. The experiment was repeated three times. Third instar larvae with the Hsp70 promoter driven human 14-3-3γ were collected after heat shocking at 37°C and allowed to recover as indicated in the figure. Total proteins were extracted and immunoblotted for presence of the human 14-3-3γ protein using a pan-specific anti-14-3-3 antibody (B and C). β-tubulin was used as loading controls in B) and C). The abbreviations depict the following: A) HS, heat-shock treatment; *yw*, *yw*^67*C*2 ^(control); *h1433GA/GC*, human 14-3-3 transgenic animals (h14-3-3A+C); 1× (heat-shocked once); 2× (heat-shocked twice); 3× (heat-shocked third time); *h1433γ*, human *14-3-3γ *primers; *rp49*, a loading control (Drosophila ribosomal protein encoding gene); RT, recovery time.

To determine the role of human 14-3-3γ (h14-3-3γ) in cell cycle regulation, we generated transgenic flies with *h14-3-3γ *cDNA inserted into a P-element vector and driven by a heat shock promoter. The h14-3-3γ transgene copy number was increased by crossing two independent lines as described in the Methods section to generate the *HS1433GA/GC *line. We first examined the *HS1433GA/GC *line for molecular evidence of h1433γ expression by performing RT-PCR and Western analyses (Figure 1). The transgenic line *HS1433GA/GC *showed h14-3-3γ mRNA expression when heat shock was applied to third instar larvae. Applying the heat shock treatment two times produced the most abundant expression of h14-3-3γ (Figure 1A; Lanes 3–5). No h14-3-3γ mRNA was observed in the parental *yw*^67*C*2 ^animals (Figure 1A; Lane 1). However, we did observe faint expression of h14-3-3γ in those transgenic lines without a heat-shock treatment which we interpreted as expression due to leakiness of the heat shock promoter which has also been observed by others [28].

We next optimized the conditions used for induction of h14-3-3γ protein. We found that a one hour heat shock treatment consistently resulted in robust induction of h14-3-3γ protein, whereas with a 30 minute heat-shock the amount of protein expressed was weak (Figures 1B &1C). Importantly, heat shock had no effect on expression of the endogenous Drosophila ε and ζ 14-3-3 genes (data not shown). Recovery time was also an important determinant for maximizing the h14-3-3γ protein expression. We found that h14-3-3γ protein expression was most highly elevated 1–3 hours after a one hour heat shock treatment (Figures 1B; Lane 6 &1C; Lanes 4–5). Neither the exogenous h14-3-3γ protein nor endogenous 14-3-3 protein could be detected in the control flies. This may be caused by the fact that only human 14-3-3γ protein could be detected in the transgenic line by using a pan-specific 14-3-3 antibody, which was raised against human 14-3-3β protein (Figure 1). Since the levels of h14-3-3γ protein declined to near background 4.5 hours after treatment (Figure 1C; Lane 5) all animals exposed to heat shock were examined within 1–3 hours after treatment.

### H14-3-3γ stimulates abnormal cell proliferation in eye imaginal discs

**Figure 2:**
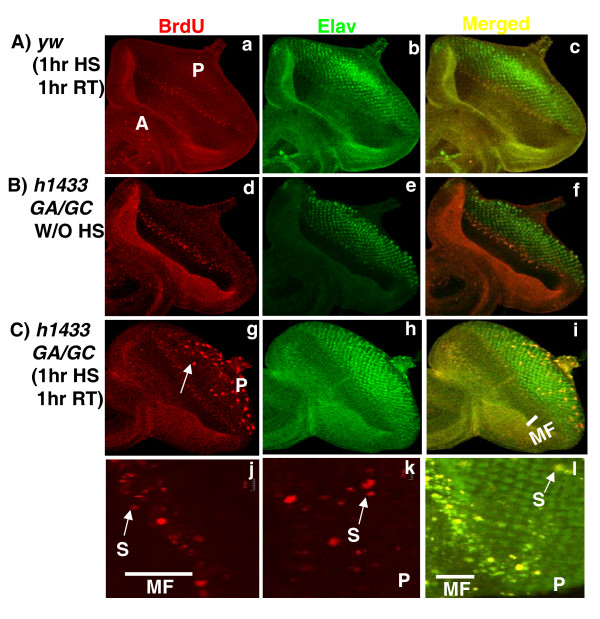
**Human 14-3-3γ stimulates abnormal proliferation in eye imaginal discs**. Third instar larvae were either heat shocked as indicated or left untreated and the eye imaginal discs dissected and processed to incorporate BrdU as described in the methods section. A mouse anti-BrdU monoclonal antibody was used to detect BrdU-labeled cells. An anti-*Elav *antibody was used to stain differentiating proneural photoreceptor cells in the region posterior to the morphogenetic furrow. Confocal images are depicted. BrdU incorporation marks replicating cells (BrdU), Elav expression signifies differentiated proneuronal cells (Elav). Merged images show the relative placement of replicating cells relative to differentiating cells (Merged). In photomicrograph "a" the letter A marks the anterior region of the eye disc. The letter P marks the posterior region of the disc. In photomicrograph "g" a white arrow points to replicating cells in the posterior compartment of the eye imaginal disc. The letter P marks the posterior compartment of the disc. In photomicrograph i "MF" marks the morphogenetic furrow (the white bar). In "J" and "K" panels, BrdU-incorporated "S" phase cells were shown (white arrows) in the 2^nd ^mitotic wave region ("J") behind of morphogenetic furrow and in the differentiating proneuronal cell region ("K"), respectively. In photomicrograph"l", a combined image of anti-BrdU and anti-elav stained cells is shown. The white scale bars, on the right top corners in the photomicrographs "J" and "K", show 20 μm.

In human lung cancer cell lines, h14-3-3γ may interfere with normal cell cycle progression [23]. To determine whether overexpression of 14-3-3γ had any effect on cell proliferation we examined the differentiating neuronal cells in the posterior compartment of the developing eyes in *HS1433GA/GC *larvae that had been heat shocked. BrdU incorporation was used to identify S-phase cells and immunostaining with anti-*Elav *antibody marked differentiating neuronal cells. We found BrdU-incorporated cells in the anti-*Elav *(neuronal cell marker) staining positive regions in *HS1433GA/GC *imaginal discs after a heat shock treatment (Figure 2C). No replicative cells were seen in the *Elav*-staining regions of eye discs from animals that were not heat shocked or in the control animals (Figures 2A &2B). The number of replicative cells, determined through BrdU incorporation, in the anti-*Elav *positive region was quantitated in control and transgenics and the results presented in Table 1. The measurements showed that the average number of S phase cells in the *Elav *positive region of *HS1433GA/GC *imaginal discs increased significantly when the larvae were heat shocked compared to animals from the same line but not heat shocked. There was small increase in BrdU-incorporating cells in *HS-h1433γ *discs without heat shock compared to *yw*^67*C*2 ^control flies. This is probably due to leakiness of the Hsp70 promoter (Figure 1A; Lane2). Our data shows that overexpression of h14-3-3γ promotes abnormal cell proliferation in differentiating tissue and that the effect is dose dependent.

**Table 1:** Frequency of BrdU incorporation in the posterior region of morphogenetic furrow in *h14-3-3γ *transgenic lines

Flies bearing transgene h14-3-3γ *	Heat-shock induction of transgene	Numbers of cells in "S" phase	Numbers of eye imaginal discs observed
None (Control)			
- HS	NO	4.14 (± 1.03)	8
+ HS	NO	8.29 (± 3.11)	8
*h14-3-3γ*			
- HS	NO	9.90 (± 1.92)	11
+ HS	YES	11.0 (± 5.69)	3
*Both HS1433GA & HS1433GC*			
- HS	NO	8.0 (± 1.91)	10
+ HS	YES	20.5 (± 2.32)^a^	11

- HS (without a heat-shock treatment)

+ HS (with 1 hr heat-shock and 1 hr recovery time)

* *HS1433GA *– on X-chromosome, homozygous or hemizygous

*HS1433GC *– on 3^rd ^chromosome, homozygous

^a ^p-value of 0.006 when compared with controls

### H14-3-3γ protein controls S phase cell prolongation

Our experiments with the Hsp70 promoter-driven *h14-3-3γ *gene suggested that the overexpression of the 14-3-3γ resulted in aberrant cellular proliferation. To confirm our results we made additional flies in which h14-3-3γ expression was specifically targeted to behind of the morphogenetic furrow of posterior compartment of 3^rd^-instar eye imaginal discs using a *GMR (Glass Multiple Reporter)-Gal4 *[29] driver. Transgenic *UAS-h1433 *γ (on 2^nd ^chromosome, #15D) flies were created as described in the Methods section. Crossing Gal4 flies with *UAS-h1433 *γ flies induced expression of h1433γ in eye imaginal discs.

**Figure 3:**
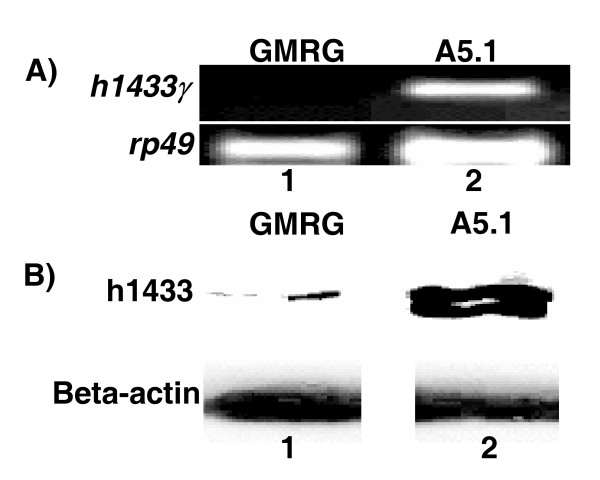
**Overexpression of human 14-3-3γ mRNA in Drosophila eye tissue using the *GMR-Gal4 *driver**. (A) Third instar larvae imaginal discs were collected and total RNA isolated. Human 14-3-3γ specific primers were used for RT-PCR to amplify human 14-3-3γ. PCR products were elecrophoresed on agarose gels and bands detected by ethidium bromide staining. *Drosophila *rp49 (coding for *Drosophila *ribosomal protein 49) was used as a loading control for RT-PCR. (B) Imaginal discs were collected as in A and total protein extracted. Immunoblotting was used to detect 14-3-3γ protein using a pan-specific anti-14-3-3 antibody. Beta-actin was utilized as a loading control. GMRG refers to the control, *GMR-Gal4 *driver only (lanes 1) and A5.1 strain has the GMR-GAL4 driven human 14-3-3γ cDNA (Lanes 2).

To mark the compartment posterior to the morphogenetic furrow in the 3^rd^-instar larval eye imaginal discs a GFP (Green Fluorescent Protein) reporter gene was introduced which also responded to the *GMR-Gal4 *driver. To induce expression of h14-3-3γ, females containing the *GMR-Gal4 *driver carrying with h14-3-3γ were crossed with male *UAS-GFP *flies (see Methods section) to create the y*w; GMR-Gal4, UAS-h1433γ/UAS-GFP *genotype. We first confirmed that h14-3-3γ expression was occurring using RT-PCR and Western blotting to detect expression (Figure 3). A PCR product of the predicted size of human 14-3-3γ mRNA was detected in these larvae indicating the presence of human 14-3-3γ mRNA (Figure 3A; Lane 2), and no product was detected in the control (Figure 3A; Lane 1). Moreover, examination of h14-3-3γ protein levels showed that the exogenous human protein was expressed in the y*w; GMR-Gal4, UAS-h1433γ/UAS-nlsGFP *transgenics (Figure 3B; Lane 2), and not in the controls.

**Figure 4:**
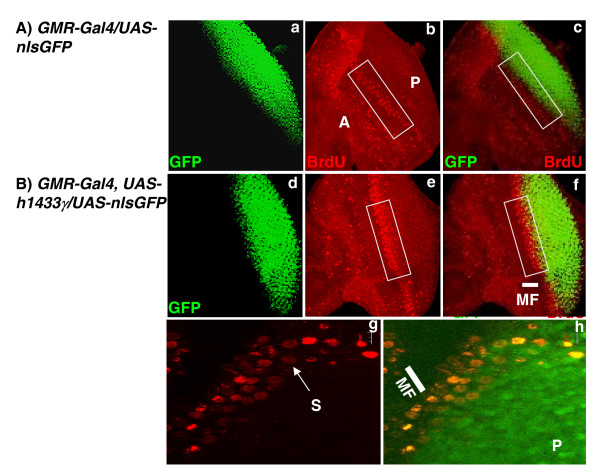
**Human 14-3-3γ induces increased cell proliferation in the morphogenetic furrow region of eye imaginal discs**. Third instar larvae were collected and processed for incorporation of BrdU as described previously and the imaginal discs dissected and examined using confocal microscopy. GFP expression (GFP) is shown in green and is due to the presence of *UAS-nlsGFP*. BrdU incorporation (BrdU) is shown in red. Merged images are shown on the right. (A) Shows control animals (*w; GMR-Gal4/UAS-nlsGFP*). (B) Shows imaginal disc from transgenic line (*yw; GMR-Gal4, UAS-h1433γ/UAS-nlsGFP*). In photomicrograph "b" the letter P marks the posterior side of the imaginal disc and the letter A marks the anterior side. In photomicrographs "b" and "e" white rectangular boxes mark the position of the second mitotic wave. MF (the white bars, "f" and "h") marks morphogenetic furrow. The letter S (the white arrow) in photomicrograph "g", refers to cells in "S" phase. The letter P marks posterior compartment of the eye disc ("h"). The size of white scale bars on the top right corners in photomicrographs "g" and "h" is 20 μm.

We next examined the eye imaginal discs of y*w; GMR-Gal4, UAS-h1433γ/UAS-nlsGFP *3^rd ^– instar larvae. As in the previous experiments, BrdU incorporation was used to detect replicating cells. GFP expression marked activity of the *GMR-Gal4 *driver (Figure 4). In Figure 4 panels b and e, which depict BrdU incorporation, the replicating cells marking the second mitotic wave are clearly visible as a band of cells laid out across the center of the imaginal disc. Notably, the apparent width of the SMW is greater in eye discs from flies expressing h14-3-3γ Figure 4B, f). This suggested that there is an increase in the(number of replicating cells within this region. Consistent with this, comparison of the merged images (which showed the relationship between the replicating cells in the second mitotic wave and GFP expression) indicates that replication ceases prior to the onset of GFP expression in control flies (Figure 4A). In contrast, GFP expression invades the band of replicating cells of the second mitotic wave in h14-3-3γ expressing animals (Figures. 4B, d–f). Taken together, these data suggest that h14-3-3γ overexpression may increase the number of proliferative cells or prolong the proliferative phase of cells in the developing fly eye resulting in a wider and more intense band of BrdU incorporating cells.

**Figure 5:**
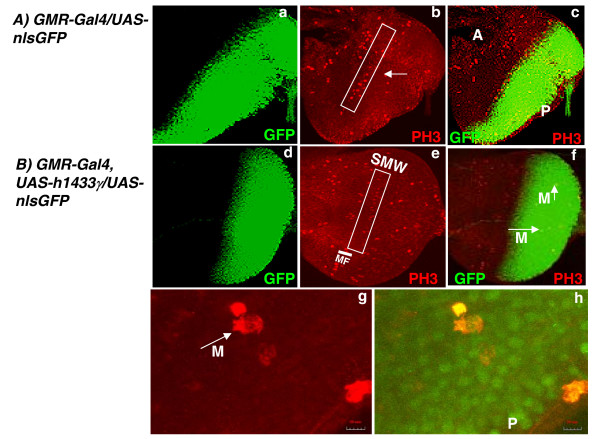
**Human 14-3-3γ suppresses appearance of mitotic cells in Drosophila eye imaginal discs**. Eye imaginal discs from third instar larva from either control (A) or transgenic (B) flies were collected and stained with an anti-Phospho-Histone H3 (PH3) antibody which detects mitotic cells. GFP expression due to the presence of *UAS-nlsGFP *is shown in green. Phospho-Histone H3 staining is shown in red. Merged images are shown at the right. In photomicrograph "b" a white arrow points to a typical mitotic cell and in "b" and "e" white rectangular boxes mark the morphogenetic furrow and the second mitotic wave. In photomicrograph "c" the position the letter A marks the anterior portion of the eye disc and the letter P marks the posterior region. In "e" panel, SMW refers to second mitotic wave. The white arrows with the letters M ("f" and "g") depict mitotic cells. The letter P in photomicrograph "h" marks the posterior compartment of the eye disc. The white scales at the bottom right corners in "g" and "h" panels show 20 μm.

We and others have suggested that 14-3-3 proteins may regulate entrance into mitosis by regulating activity of the Cdc25 phosphatase [17,30], Hence, we sought to determine whether the h14-3-3γ expression had any effect on the onset of mitosis. Consequently we examined the occurrence of mitosis in eye imaginal discs of flies expressing *GMR-Gal4*-driven h14-3-3γ. Eye imaginal discs were collected from third instar larvae and immunostained for phospho histone H3 (PH3) and the samples examined using confocal microscopy to detect PH3 and GFP (Figure 5). As can be seen mitotic cells are apparent near the morphogenetic furrow of eye imaginal discs from control animals (Figure 5A, b), but that this band of mitotic cells is markedly reduced in eye discs where h14-3-3γ is overexpressed (Figure 5B, e). This suggests that h14-3-3γ can suppress entrance into mitosis in a manner similar to that observed in animal cells [30].

To further examine the mechanism of h14-3-3γ activity we decided to overexpress h14-3-3γ in a genetic background with compromised Cdc25 (*String*). To this end, we introduced the heat shock inducible h14-3-3γ gene into a genetic background containing the *String *hypomorphic allele, *Stg*^9*A *^and then induced h14-3-3γ using the heat shock protocol described previously [31]. In the double mutant we found 8.96% (SEM: ± 1.27, 21 out of total 257 flies) of adult eyes had a rough eye phenotype (Table 2). However, in the absence of *Stg*^9*A *^we found only 1.61% (SEM: ± 1.03, 2 out of total 211 flies) displaying the phenotype. By comparison only 1.39% (3 out of total 191 flies) of the parental background (*yw*^67*C*2^) flies showed the rough eye phenotype. Hence, presence of the hypomorphic *String *allele appears to increase sensitivity to the effects of h14-3-3γ suggesting that h14-3-3γ may interact with Cdc25 in cell cycle regulation.

**Table 2:** Induction of the rough eye phenotype by *h14-3-3γ *in a heterozygous *String *genetic background

Flies bearing transgene *h14-3-3γ*	*Stg*^9*A *^genetic background	Rough eye phenotype (%)	Numbers of flies observed
No (Control)	No (Control)	1.39	191
No (Control)	Yes (heterozygous)	0	20
Yes	No (Control)	1.61	211
Yes	Yes (heterozygous)	8.96*	257

* Statistically significant difference when compared with controls (P-value < 0.05)

## Discussion

Collectively our results suggest that overexpression of human 14-3-3γ leads to the abnormal appearance of replicating cells in eye imaginal discs where such cells would normally not appear. Although the effect was modest, the appearance of abnormally proliferative cells was reproducible when h14-3-3γ gene copy number was increased. It is unclear why the effect of h14-3-3γ overexpression was slight. One possibility is that h14-3-3γ, which likely evolved from the other isoforms, has only partially overlapping functions with the endogenous 14-3-3γ. In any case, overexpression of h14-3-3γ resulted in replicating cells appearing amongst differentiated neuronal cells posterior to the morphogenetic furrow in eye imaginal discs. These BrdU-incorporating cells could result from h14-3-3γ causing differentiated cells to become abnormally replicative or because h14-3-3γ causes replicative cells to remain in the replicative phase for a prolonged period. We favor the latter hypothesis. Progression of the morphogenetic furrow through the undifferentiated eye disc is precisely regulated. The number of replicating cells that arise in the wake of the morphogenetic furrow is tightly controlled and is typically about two cells deep. However, in the eye imaginal discs of flies where h14-3-3γ is driven by *GMR-Gal4 *the width of the band of proliferative cells is increased to between 3–4 cells. Concomitantly, we showed that h14-3-3γ suppressed the appearance of mitotic cells in the eye discs of these same flies. This is consistent with data from our lab and with what has been shown for another 14-3-3 family member 14-3-3σ [32] and could indicate that 14-3-3γ is involved in the process that prolonging replicative phase of cells delay entry into mitosis. Indeed, in previous studies we showed that 14-3-3γ caused cells to reenter S phase when overexpressed in a lung cancer cell line [23].

## Conclusion

The primary conclusion from these studies is that h14-3-3γ leads to abnormal cell proliferation when overexpressed and that proliferation is evident even after the tissue has become differentiated. This has important implications for h14-3-3γ as an oncogene as it suggests that elevated levels of the protein can interfere with normal cell cycle progression.

## Methods

### Generation of transgenic fly stocks and genetic crosses

Transgenic flies, *yw; UAS-h1433γ *and *hs-h1433γ *lines were generated using a cloned human 14-3-3γ cDNA [23] into P-element vectors such as pUASP [33] and pCaSpeR-*hs *[34], respectively.

For ubiquitous h14-3-3γ protein expression, we generated two transgenic lines that express gamma using a P-element vector in which the *h14-3-3γ *gene was driven by the Hsp70 heat shock promoter and could be activated by heat shock. Two independent lines were generated, *HS1433GA *(on X chromosome) and *HS1433GC *(on 3^rd ^chromosome). To increase gene copies (more than 2) of h14-3-3γ progeny, these flies were crossed with each other and selected using eye color as a marker for gene dosage. A stock homozygous for 14-3-3γ on the X and 3^rd ^chromosome was established from this cross and has remained stable for an extended period of time.

Stock flies with *GMR-Gal4 *driver were crossed with 8 *UAS-h14-3-3γ *transgenic lines for initial screening, and we found all lines to be similar. For the experiments in this study, male flies of *h14-3-3γ *on the 2^nd ^chromosome (P33#15D) were crossed with *GMR-Gal4 *(on 2^nd ^chromosome) female flies to generate recombinant flies for the experiments and scored for eye phenotype. Recombinant flies bearing both *UAS-h14-3-3γ *and *GMR-Gal4 *on the second chromosome were balanced with CyO balancer chromosome. Virgin female homozygous *GMR-Gal4*, *UAS-h14-3-3γ *flies were selected to cross with homozygous male *UAS-GFP *(2^nd ^chromosome) flies (Figures 4 and 5).

Using those increased dosage of *hs-1433γ *flies,. we crossed on h14-3-3γ transgenes into a *String *mutant genetic background. *String *is a homologue of Cdc25, and the allele of *Stg*^9*A *^(a kind gift from Dr. Patrick O'Farrell) is known to be temperature-sensitive. On the 3^rd ^chromosome, *Stg*^9*A *^is balanced with TM3 (third multiple 3) having Sb^' ^(Stubble) as a dominant marker [35]. To examine eye phenotypes in adults expressing h14-3-3γ in *Stg*^9*A *^heterozygotes, we treated the flies with multiple heat-shocks (a 30 min heat pulse at 37°C every 7 and a half hours) starting from 3^rd^-instar larval stage.

### RT-PCR

RT-PCR was performed using total RNA extracted from adult flies or 3^rd^-instar larvae. For the total RNA extraction, a FastRNA Pro Green kit (Qbiogene) was used and followed the manufacturer's instructions. For Reverse Transcription (RT), a mixture of Oligo-dT, dNTPs (10 mM), RNA (5 μg), and DEPC- ddH2O was incubated for 5 min at 6°C. The RT contents were collected at the bottom by centrifuging, and 5 × buffer, 0.1 M DTT and RNAse inhibitor were added. After they were incubated for 2 minutes at 42°C, 1 μl Superscript II RT was added. For PCR reactions, a mixture of 41 μl Platinum Supermix (Invitrogen), 3 μl DMSO, 10 mM dNTP, DNA Digest 1 (1 μg of Total RNA, 10× DNAase free buffer, DNAase, ddH2O up to 10 μl) and 10 μM each forward and reverse primer for 1433γ cDNA were added (Forward, CTGAATGAGCCACTGTCGAA; Reverse, CACACAGCCTCCAACTCCTT). *Drosophila *ribosomal protein 49 encoding gene (*dRP49*) was used as a loading control for the PCR reactions. The primer sequences to flank *dRP49 *were 5'-GTGTATTCCGACCACGTTACA (RP49-antisense) and 5'-TCCTACCAGCTTCAAGATGAC (RP49-sense).

### Western blotting

Cell lyses were performed on ice by using 3^rd^-instar larval imaginal discs in RIPA buffer. Total protein 50 μg per lane was loaded on a SDS/PAGE gel. The protein bands were transferred onto Nitrocellulous membranes (BioTrace NT, Pall Corporation) in transfer buffer (89.3 g glycine, 19.3 g Tris, 1.6 L Methanol, ddH_2_O up to 8 L) for overnight on a Transphor Unit (Amersham Biosciences, Cat # 80-6205-97). Then, a primary antibody, mouse anti-14-3-3β antibody (pan-specific antibody detecting all human 14-3-3 isoforms, Santa Cruz, Cat # SC1657), was used at a 1:100 dilution at 4°C for overnight. A goat anti-mouse secondary antibody was diluted 1:5000 in PBST (1 × PBS + 0.1% Tween 20) buffer with 5% Non-Fat Dry Milk, and the membrane was incubated in the secondary antibody for 2 hrs at room temperature. Using an ECL detection kit (Pierce), specific protein bands were detected onto X-ray films (Kodak) using an autoradiographic machine (Konica SRX-101A). The membranes were stripped in 0.1 M NaOH for 5 min at room temperature and treated with a loading control anti-beta-actin antibody(AbCam).

### Immunostaning

Mature 3^rd^-instar larvae were collected from controls (Wild Types) and 1433γ overexpressing animals. Heat-shock treatment was performed for 1 hr at 37°C in a water bath. After the heat-shock treatment, 1 hr of recovery time was given. For fixation, imaginal discs were dissected and immediately treated with 5% paraformaldehyde for 30 min. Tissues were washed twice for 10 min. in PBST (1 × PBS, 0.1% Triton ×-100). For a blocking step, 1% Normal Goat Serum (NGS) was added to PBST and treated for 30 min. A neuronal cell marker, rat anti-elav antibody was diluted 1:200. An anti-pH3 (phosphorylated Histone H3 at Serine 10, Upstate Biotechnology) antibody was used at 1:250 dilution.

The primary antibodies were added and incubated for overnight at 4°C. The antibody rat anti-elav was obtained from the Developmental Studies Hybridoma Bank at the University of Iowa. FITC- or TRITC-labeled secondary antibodies (KPL) were treated for 2 hr at room temperature. After washing in PBS-T and PBS-BT (PBST + 0.5% BSA), the tissues were mounted in Mowiol mounting medium (Calbiochem, Cat # 475904).

### BrdU incorporation

The BrdU (Bromodeoxyuridine) incorporation was performed in 1 × PBS with 5 μg/ml BrdU for 1 hr at room temperature with gentle shaking on Nutator. Imaginal discs were fixed in 5% paraformaldehyde for 30 min, and washed for 5 min 3× in PBST (1 × PBS, 0.3% Triton ×-100). Then, the tissues were treated with 2 N HCl for 30 min and neutralized for 2 min in 100 mM Borax (Sigma). A primary antibody, mouse anti-BrdU (Becton Dickinson) was used at a 1:20 dilution in a mixture of PBST and 5% NGS (Vector Labs). The primary antibody incubation was done at 4°C overnight. A goat-anti mouse TRITC-labeled secondary antibody (Jackson ImmunoResearch) was used at a 1:200 dilution and the imaginal discs were treated for 2 hrs at room temperature. The discs were mounted using Vectashield mounting medium (Vector Labs).

### Confocal laser scanning and fluorescence microscopic studies

Confocal images were collected by using a Confocal Microscope (Nikon Eclipse E800). For screening of the immunostaining, a fluorescence microscope (Nikon Eclipse E800) with X-cite 120 (Fluorescence Illumination Systems) was also utilized. The microscopic images were analyzed by using an Adobe Photoshop 7 software.

## Competing interests

The author(s) declare that they have no competing interests.

## Authors' contributions

SWH and JDM designed the experiments and analyzed the data after SWH performed the experiments. SWH and JDM also worked on the manuscript and JDM edited the manuscript as a principle investigator. WQ generated vector constructs for fly transformation. MB performed Fly Transformation for the transgenic lines. GB gave advice on the experiments and manuscript as a co-principle investigator with JDM. All authors discussed, read and approved the final version of manuscript.
